# Luseogliflozin attenuates neointimal hyperplasia after wire injury in high-fat diet-fed mice via inhibition of perivascular adipose tissue remodeling

**DOI:** 10.1186/s12933-019-0947-5

**Published:** 2019-10-31

**Authors:** Yusaku Mori, Michishige Terasaki, Munenori Hiromura, Tomomi Saito, Hideki Kushima, Masakazu Koshibu, Naoya Osaka, Makoto Ohara, Tomoyasu Fukui, Hirokazu Ohtaki, Hirano Tsutomu, Sho-ichi Yamagishi

**Affiliations:** 1 Division of Diabetes, Metabolism, and Endocrinology, Department of Medicine, Showa University School of Medicine, Tokyo, 142-8555 Japan; 2 Diabetes Center, Ebina General Hospital, Kanagawa, 243-0433 Japan; 3 Department of Anatomy, Showa University School of Medicine, Tokyo, 142-8555 Japan

**Keywords:** Arterial remodeling, Obesity, Perivascular adipose tissue, SGLT2 inhibitor

## Abstract

**Background:**

Excess fat deposition could induce phenotypic changes of perivascular adipose tissue (PVAT remodeling), which may promote the progression of atherosclerosis via modulation of adipocytokine secretion. However, it remains unclear whether and how suppression of PVAT remodeling could attenuate vascular injury. In this study, we examined the effect of sodium-glucose cotransporter 2 (SGLT2) inhibitor, luseogliflozin on PVAT remodeling and neointima formation after wire injury in mice.

**Methods:**

Wilt-type mice fed with low-fat diet (LFD) or high-fat diet (HFD) received oral administration of luseogliflozin (18 mg/kg/day) or vehicle. Mice underwent bilateral femoral artery wire injury followed by unilateral removal of surrounding PVAT. After 25 days, injured femoral arteries and surrounding PVAT were analyzed.

**Results:**

In LFD-fed lean mice, neither luseogliflozin treatment or PVAT removal attenuated the intima-to-media (I/M) ratio of injured arteries. However, in HFD-fed mice, luseogliflozin or PVAT removal reduced the I/M ratio, whereas their combination showed no additive reduction. In PVAT surrounding injured femoral arteries of HFD-fed mice, luseogliflozin treatment decreased the adipocyte sizes. Furthermore, luseogliflozin reduced accumulation of macrophages expressing platelet-derived growth factor-B (PDGF-B) and increased *adiponectin* gene expression. Gene expression levels of *Pdgf*-*b* in PVAT were correlated with the I/M ratio.

**Conclusions:**

Our present study suggests that luseogliflozin could attenuate neointimal hyperplasia after wire injury in HFD-fed mice partly via suppression of macrophage PDGF-B expression in PVAT. Inhibition of PVAT remodeling by luseogliflozin may be a novel therapeutic target for vascular remodeling after angioplasty.

## Background

Cardiovascular disease (CVD) is a leading cause of death in many countries [1]. Obesity is one of the representative risk factors for CVD, and phenotypic change of visceral adipose tissue (VAT), known as VAT remodeling, is a causal factor that could link visceral obesity to CVD [2, 3]. In response to various metabolic derangements, VAT secrets numerous kinds of adipokines, which can exert cardiovascular actions, thereby being involved in CVD [4–6]. Indeed, excess fat deposition in VAT further promotes the metabolic derangements by altering adipokine profiles, which results in the development and progression of CVD [5, 6].

Recently, perivascular adipose tissue (PVAT) has gained attention as a potential modifiable risk factor for CVD [7, 8]. PVAT have traditionally been regarded as tissues merely providing mechanical support for the vasculature. However, accumulating evidence has shown that PVAT actively secrets various adipokines, and ectopic fat deposition in PVAT could stimulate the remodeling of this tissues and cause the alteration of adipokine secretions as is the case in VAT [9, 10]. Furthermore, a couple of papers have suggested the involvement of PVAT remodeling in atherosclerosis in rodent models [11, 12]. However, the underlying mechanisms are not fully elucidated. In addition, it remains also unclear whether suppression of PVAT remodeling could attenuate the vascular injury and progression of atherosclerosis in mice.

Sodium-glucose cotransporter-2 (SGLT2) is an apical membrane-bound transporter, which is selectively expressed at S1 segment of renal proximal tubular epithelial cells, being involved in urinary glucose reabsorption [13, 14]. SGLT2 inhibitors are one of the widely used oral hypoglycemic agents because inhibition of SGLT2 attenuates hyperglycemia by increasing urinary glucose excretion, which is associated with reduction of blood pressure, body weight, waist circumstance, and VAT [15–17]. Furthermore, several papers have shown that SGLT2 inhibitors could also reduce ectopic fat deposition in the other VATs, such as hepatic and pericardial adipose tissues [18–21]. The findings led us to speculate that inhibition of SGLT2 may become a therapeutic approach to attenuate the remodeling of PVAT.

In this study, we examined the effect of luseogliflozin, a highly selective SGLT2 inhibitor [22] on PVAT remodeling and neointimal formation after wire injury in high-fat or low-fat fed mice in order to address the issues whether and how suppression of PVAT remodeling could protect against vascular injury in animal models.

## Materials and methods

### Animal studies

The study design was approved by the Animal Care Committee of Showa University School of Medicine (approval numbers: 09059). Animal experiments were conducted under strict adherence to the Guide for the Care and Use of Laboratory Animals [23]. All invasive procedures were performed under general anesthesia using isoflurane. C57BL/6J (wild-type) mice were purchased from Sankyo Labo Service (Edogawa, Tokyo, Japan). Mice were maintained on standard rodent chow (Labo MR Stock, NOSAN, Yokohama, Kanagawa, Japan) with free access to water and housed within a specific pathogen-free facility in the Division of Animal Experimentation of Showa University School of Medicine. The rooms were controlled under a 12-h dark/light cycle, 21 °C, and 40–60% humidity.

Male wild-type mice at the age of 8 weeks were switched to feeding with high-fat diet (HFD) or low-fat diet (LFD), which were composed of 45% or 10% kcal from fat (D12451 and D12450H, Research Diets, New Brunswick, NJ, USA), respectively. On Day 60, mice were assigned to treatment with each diet containing vehicle or luseogliflozin (ca. 18 mg/kg/day) [24, 25]. Luseogliflozin was provided by Taisho Pharmaceutical Co., Ltd. (Toshima, Tokyo, Japan). On Day 75, mice were subjected to bilateral femoral artery wire injury followed by unilateral removal of surrounding PVAT as described previously with a few modifications [26]. In brief, a straight spring wire [C-SF-15-15; Cook Japan, Nakano, Tokyo, Japan] was inserted in a retrograde manner into the femoral artery via a small cut on the muscle branch, and withdrawn after 1 min. The wire insertion was repeated once, and the muscle branch was ligated proximal to the cut to prevent bleeding. In the left femoral artery, surrounding PVAT was gently removed by fine forceps. After the procedure, the skin incision was sutured. On Day 100, tissue samples were collected for morphological, immunohistochemical and reverse-transcription polymerase chain reaction (RT-PCR) analyses. Fat and liver indexes were calculated by dividing epididymal fat pad and liver weights by body weight, respectively.

### Measurement of plasma biochemistry

Blood samples were collected after 6 h-fasting at the end of each experiment. Plasma glucose levels were determined using Nipro Statstrip XP2 (Nipro, Osaka, Osaka, Japan). Plasma lipid parameters were assessed using an enzymatic colorimetric assay (Fujifilm Wako Pure Chemical, Osaka, Osaka, Japan). Plasma levels of insulin and glucagon were measured by enzyme-linked immunosorbent assays (Ultra Sensitive “PLUS” Mouse Insulin ELISA Kit, Product ID M1105; Morinaga, Yokohama, Kanagawa, Japan; Glucagon ELISA Kit, Product ID 292-90001; Fujifilm Wako Pure Chemical).

### Assessment of blood pressure

Systolic blood pressure and pulse rates were measured using a tail-cuff method at 3 to 5 days prior to the end of each experiment (Model MK-2000ST; Muromachi Kikai, Chuo, Tokyo, Japan) [27]. The measurement was conducted between 11:00 AM to 3:00 PM under conscious conditions. The average value obtained from several consecutive measurements was used as a single data point.

### Morphometric analysis

Collected femoral arteries and epididymal adipose tissue were embedded into paraffin blocks, and their cross-sections were stained with Elastica van Gieson or hematoxylin and eosin (H&E). In the assessment of femoral arteries, average values of several serial cross-sections were used as a single data point to minimize the selection bias as described previously [27]. Neointima and media were defined as the areas between the limen and internal elastic lamina and between the internal and external elastic lamina, respectively. The data of following sections were excluded from the present study: sections with large thrombus burden and occlusion, broken vascular wall structure, a branch of another artery, and missing elastic lamina [27]. The histological assessment was conducted by an independent investigator who was blinded to the treatment conditions using ImageJ software [28].

### RT-PCR analysis

Total RNA was extracted from tissues with Isogen reagent (NIPPON GENE, Chiyoda, Tokyo, Japan) and then reverse-transcribed to cDNA with ReverTra Ace qPCR RT Kit (Toyobo, Osaka, Osaka, Japan) according to the manufacturer’s instructions. Gene expression was assessed by using the TaqMan gene expression assay and sequence detection system (StepOne Plus; Life Technologies Japan, Minato, Tokyo, Japan) as previously described [27]. The pre-designed TaqMan probe sets used were as follows: *Adiponectin*, Mm00456425_m1; interleukin-1b (*Il*-*1b*), Mm00434228m1; *Il*-*6*, Mm00446190_m1; monocyte chemotactic protein-1 (*Mcp*-*1*), Mm00441242_m1; platelet-derived growth factor-B (*Pdgf*-*b)*, Mm00440678_m1; *Pdgf receptor*-*b*, Mm00435553_m1, tumor necrosis factor-α (*Tnf*-*α*), Mm00443258_m1. The 18S ribosomal RNA probe (*18srna*, Mm03928990_g1) was used as an internal control.

### Immunohistochemical analysis

Cross-sections were obtained from paraffin-embedded femoral arteries and then incubated with anti-F4/80 antibody (Abcam Japan, Chuo, Tokyo, Japan; Ab204467; RRID: AB_2810932; raised in rat; 1: 50) and anti-PDGF-B antibody (Abcam Japan, Ab23914; RRID: AB_2162180, raised in rabbit, 1:50) overnight. The antibodies were diluted with Antibody Diluent (S3022; Dako, Santa Clara, CA, USA). Nuclei were stained with 4′,6-diamidino-2-phenylindole (D1306; Thermo Fisher Scientific, Waltham, MA USA). The immunofluorescence images were obtained by a confocal microscope (BZ-X710 microscope; Keyence, Osaka, Osaka, Japan).

### Statistical analysis

Data are expressed as mean ± standard deviation. Statistical comparisons were performed using an unpaired *t* test for two groups and one-way or two-way ANOVA followed by Tukey’s test for three or more groups. The Pearson correlation coefficient was used to test correlations between variables. Statistical calculations were performed using JMP software (version 13; SAS Institute Inc., Cary, NC, USA). The significance level was defined as *p* < 0.05.

## Results

### Luseogliflozin treatment or PVAT removal decreased neointimal hyperplasia in HFD-fed mice

First, we evaluated the effects of luseogliflozin and PVAT removal on vascular injury in mice fed with HFD or LFD. Anthropometric and biochemical parameters are presented in Table 1. HFD-fed mice showed higher body weights than LFD-fed mice throughout the experimental periods. Fat index, glycated hemoglobin (HbA1c), plasma glucose, insulin, total cholesterol, high-density lipoprotein (HDL)-cholesterol, and triglycerides were significantly higher in HFD-fed mice compared with LFD-fed mice. Luseogliflozin significantly attenuated body weigh increase in HFD-fed mice, whereas it increased food and water intake, and plasma total cholesterol levels. On the other hand, in LFD-fed mice, luseogliflozin significantly decreased food intake, while it increased plasma HDL-cholesterol and triglycerides.

**Table 1:** Clinical parameters of HFD- and LFD-fed mice treated with vehicle or luseogliflozin

	HFD		LFD	
	Vehicle	Luseogliflozin	Vehicle	Luseogliflozin
Number	12	8	5	5
Food intake (g/day)	3.2 ± 0.3^a^	4.1 ± 0.5^bc^	4.8 ± 0.9	2.9 ± 0.2^a^
Water intake (g/day)	3.0 ± 0.3^ab^	6.0 ± 0.7^c^	7.6 ± 2.5	6.6 ± 0.4
Initial body weight (g)	40.5 ± 2.7^ab^	43.6 ± 5.1^ab^	28.7 ± 1.3	27.6 ± 2.3
Final body weight (g)	44.0 ± 2.5^ab^	45.5 ± 5.7^ab^	30.2 ± 2.2	28.2 ± 2.0
Body weight increase (g)	3.4 ± 0.9^ab^	1.8 ± 1.2^c^	1.5 ± 0.9	0.6 ± 1.0
Liver index (mg/g)	40.3 ± 7.8	38.1 ± 8.0	43.4 ± 4.1	44.1 ± 2.8
Fat index (mg/g)	59.8 ± 5.4^ab^	57.6 ± 9.8^ab^	7.5 ± 1.1	9.8 ± 2.1
Pulse (beat/min)	716 ± 70	733 ± 23	746 ± 32	709 ± 45
Systolic blood pressure (mmHg)	125 ± 6	132 ± 8^ab^	111 ± 14	112 ± 13
HbA1c (%)	5.1 ± 0.2^b^	5.0 ± 0.3	5.1 ± 0.1	4.8 ± 0.1
Plasma glucose (mg/dl)	186 ± 33^ab^	168 ± 20^a^	87 ± 12	127 ± 19
Plasma insulin (ng/ml)	0.33 ± 0.20^ab^	0.25 ± 0.17	0.04 ± 0.01	0.07 ± 0.05
Plasma glucagon (pmol/l)	7.5 ± 5.8	7.3 ± 5.0	4.2 ± 2.4	7.5 ± 4.7
Plasma total cholesterol (mg/dl)	177 ± 10^ab^	199 ± 21^abc^	78 ± 8	93 ± 7
Plasma HDL-cholesterol (mg/dl)	107 ± 6^ab^	111 ± 4^ab^	62 ± 8	75 ± 6^a^
Plasma triglycerides (mg/dl)	65 ± 10^a^	72 ± 9^a^	49 ± 3	68 ± 8^a^
Plasma free fatty acid (mEq/l)	0.50 ± 0.05	0.51 ± 0.03	0.48 ± 0.12	0.53 ± 0.06

Values are shown as mean ± standard deviation

HbA1c glycated hemoglobin *HDL* high-density lipoprotein

One-way ANOVA followed by Tukey’s test: ^a^*p* < 0.05 vs. LFD-Vehicle

^b^*p* < 0.05 vs. LFD-Luseogliflozin

^c^*p* < 0.05 vs. HFD-Vehicle

**Fig. 1:**
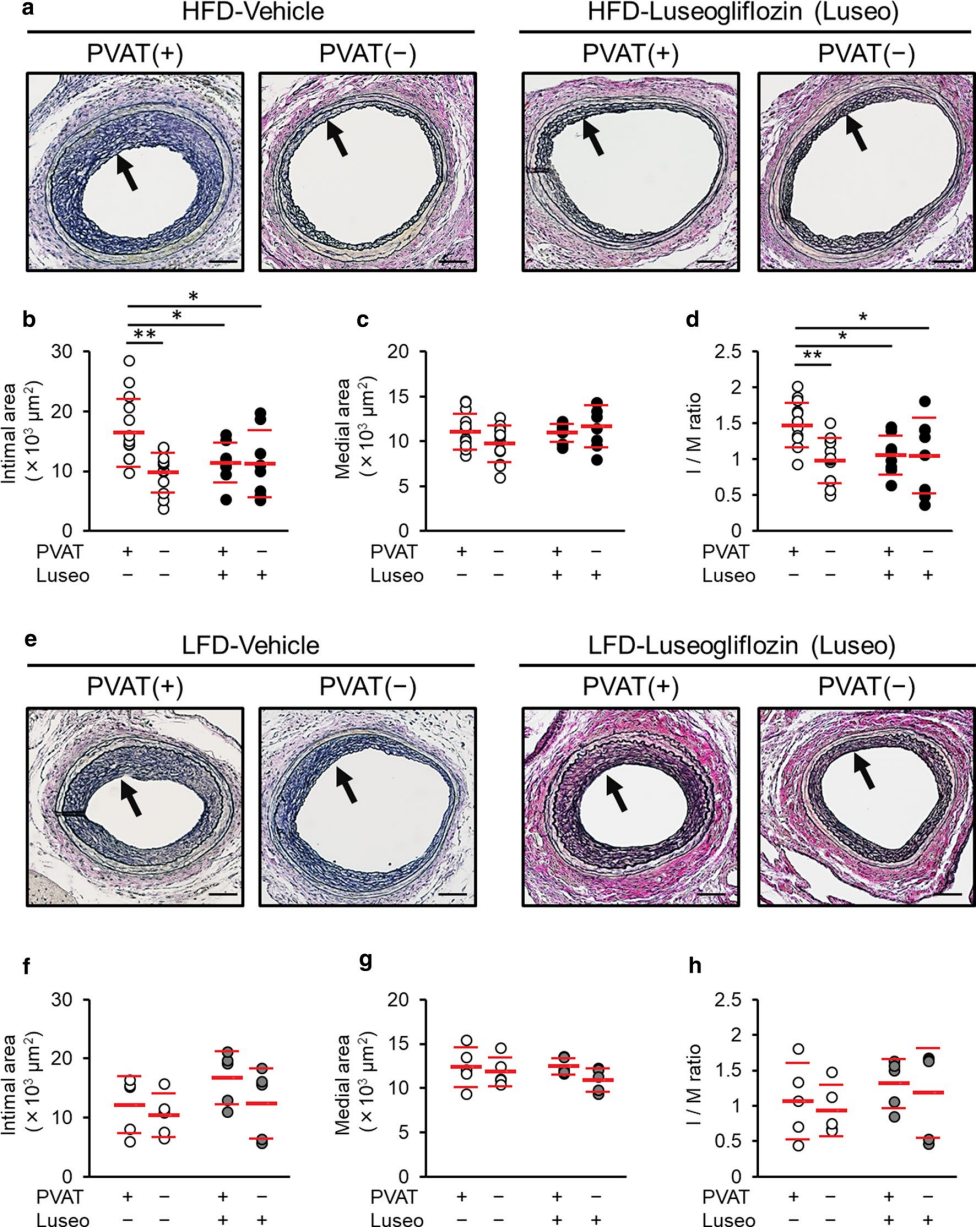
Effects of luseogliflozin and PVAT removal on vascular injury in HFD- or LFD-fed mice. HFD- or LFD-fed mice were treated with vehicle or luseogliflozin, and underwent bilateral femoral artery wire injury followed by unilateral PVAT removal. Femoral arteries were evaluated at 25 days after vascular injury. **a**, **e** Representative microscopic images of Elastica van Gieson-stained cross-sections of femoral arteries of mice fed with HFD (**a**) or LFD (**e**). Arrows indicate the neointima. Scale bar = 200 μm. **b**, **f** Neointimal area. **c**, **g** Medial area. **d**, **h** I/M ratio. **b**–**d** HFD and vehicle, *N *= 12; HFD and Luseogliflozin (Luseo), *N* = 8. **f**–**h** LFD and vehicle, *N* = 5; LFD and Luseo, *N* = 5. *p < 0.05, **p < 0.01

**Fig. 2:**
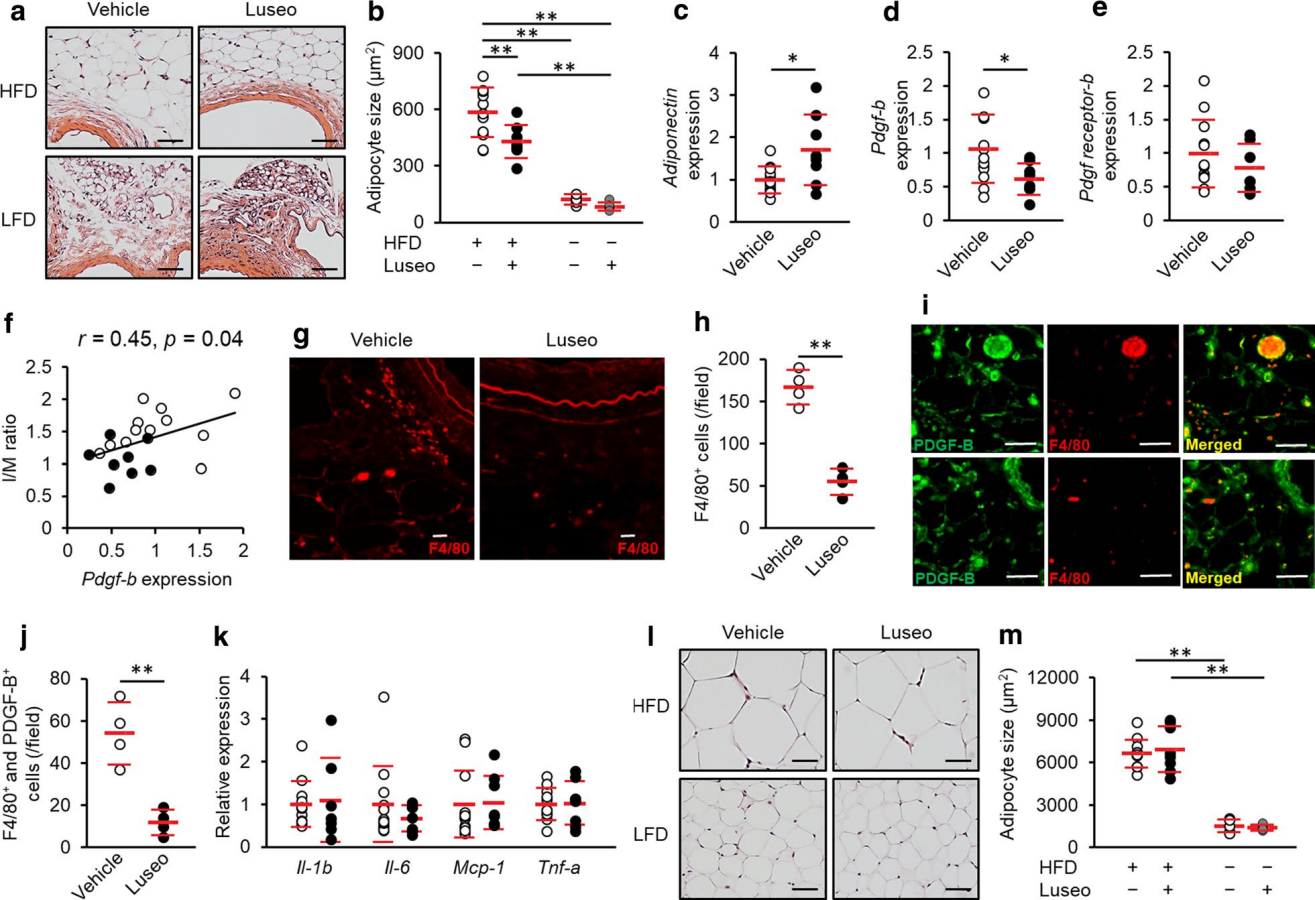
Effect of luseogliflozin on PVAT remodeling and its association with vascular injury in HFD-fed mice. PVAT surrounding the injured femoral arteries and epididymal adipose tissue at 25 days after arterial injury were collected from HFD-fed or LFD-fed mice treated with vehicle or luseogliflozin. **a** Representative images of H&E-stained cross-sections of femoral PVAT. **b** Average adipocyte size of PVAT. **c–e** Gene expression of *adiponectin* (**c**) and *Pdgf*-*b* (**d**), *Pdgf receptor*-*b* (**e**) in femoral PVAT of HFD-fed mice. **f** Correlation between *Pdgf*-*b* expression levels in femoral PVAT and I/M ratio of corresponding femoral artery. White dots, vehicle; back dots, luseogliflozin. **g** Representative images of immunofluorescence staining for F4/80 in femoral PVAT of HFD-fed mice. **h** Number of F4/80-positive cells in PVAT. **i** Representative images of immunofluorescence staining for F4/80 and PDGF-B in femoral PVAT of HFD-fed mice. Upper images, vehicle; lower images, luseogliflozin. **j** Number of cells F4/80- and PDGF-B-positive cells. **k** Relative gene expression levels of *Il*-*1b*, *Il*-*6*, *Mcp*-*1*, and *Tnf*-*α* in femoral PVAT of HFD-fed mice. White dots, vehicle; back dots, luseogliflozin. **l** Representative images of H&E-stained cross-sections of epididymal adipose tissue. **m** Average adipocyte size of epididymal adipose tissue. **a**, **g**, **i**, **l** Scale bar = 200 μm. **b**–**f**, **k**, **m**: HFD and vehicle, *N* = 12; HFD and Luseogliflozin (Luseo), *N* = 8; LFD and vehicle, *N *= 5; LFD and Luseo, *N *= 5. **h**, **j** Vehicle, *N *= 4; Luseo, *N *= 4. *p < 0.05, **p < 0.01

Morphometric changes of injured arteries 25 days after arterial wire injury are presented in Figs. 1, 2. Removal of PVAT or luseogliflozin treatment significantly reduced neointimal area and intima-to-media (I/M) ratio in control HFD-fed mice treated with vehicle (Fig. 1). However, the combination therapy of luseogliflozin and PVAT removal showed no additive effects on injured arteries (Fig. 1a–d). In LFD-fed mice, the I/M ratio was significantly smaller than that in HFD-fed mice (*p* = 0.047), whereas neither PVAT removal or luseogliflozin treatment affected the neointimal, medial area, or I/M ratio in injured arteries (Fig. 1e–h).

### Luseogliflozin attenuated the PVAT remodeling in HFD-fed mice

Next, we investigated the effects of luseogliflozin on remodeling of PVAT surrounding the injured femoral arteries. As shown in Fig. 2a, b, average size of PVAT was significantly larger in HFD-fed mice compared with LFD-fed mice, whereas luseogliflozin treatment significantly reduced the PVAT size in HFD-fed mice, but not LFD-fed mice. Luseogliflozin treatment increased *adiponectin* and decreased *Pdgf*-*b* gene expression in PVAT surrounding the injured femoral arteries of HFD-fed mice (Fig. 2c, d), whereas it did not affect *Pdgf receptor*-*b* gene expression (Fig. 2e). Gene expression levels of *Pdgf*-*b* were significantly correlated with the I/M ratios of the corresponding femoral arteries (Fig. 2f). Furthermore, immunofluorescence staining revealed that luceogliflozin treatment significantly decreased the number of infiltrated macrophages, evaluated by F4/80 staining, into the femoral PVAT of HFD-fed mice (Fig. 2g, h). In addition, luseogliflozin treatment reduced the number of PDGF-B-coexpressed F4/80-positive cells in the PVAT (Fig. 2i, j). As shown in Fig. 2k, gene expression levels of pro-inflammatory cytokines, such as *Il*-*1b*, *Il*-*6*, *Mcp*-*1*, and *Tnf*-*α* were not affected by luseogliflozin treatment. In contrast to the case in PVAT, luseogliflozin treatment did not affect the average size of epididymal adipose tissue, another VAT (Fig. 2l, m).

## Discussion

Perivascular transplantation of PVAT obtained from obese mice has been shown to promote the neointimal hyperplasia after arterial injury in this animal model [10, 29]. However, it remained unclear whether PVAT remodeling under pathological conditions, such as high-fat diet could intrinsically contribute to neointimal hyperplasia after wire injury in mice. To address this issue, we used here HFD-fed and LFD-fed mice, which were subjected to bilateral femoral artery wire injury followed by unilateral removal of surrounding PVAT. In the present study, we found for the first time that removal of the surrounding PVAT significantly suppressed the wire-injured neointimal hyperplasia in corresponding femoral artery of HFD-fed, but not LFD-fed mice. Although balloon angioplasty and endovascular stent implantation are standard clinical practices for the treatment of coronary artery disease, these therapeutic options are far from satisfactory because of higher rate of restenosis or late stent thrombosis [30, 31]. Therefore, our present observations suggest that PVAT could be crucially involved in the progression of neointimal hyperplasia after wire injury, being a novel therapeutic target for reducing the vascular damage after balloon or stent angioplasty.

In this study, we also found that as is the case in PVAT removal, oral administration of luseogliflozin significantly reduced the neointimal hyperplasia after wire injury in HFD-fed, but not LFD-fed mice. Furthermore, the beneficial effects of luseogliflozin were not observed in injured femoral artery without PVAT.

However, we did not find any effect of luseogliflozin on neointimal hyperplasia in the absence of PVAT remodeling such as PVAT-removed femoral arteries of HFD-fed mice and PVAT-intact femoral arteries in LFD-fed mice. These findings suggest that protective effects of luseogliflozin against neointimal formation after wire injury could be ascribed in part to its actions on remodeled PVAT, but not vascular cells or lipid metabolisms.

**Fig. 3:**
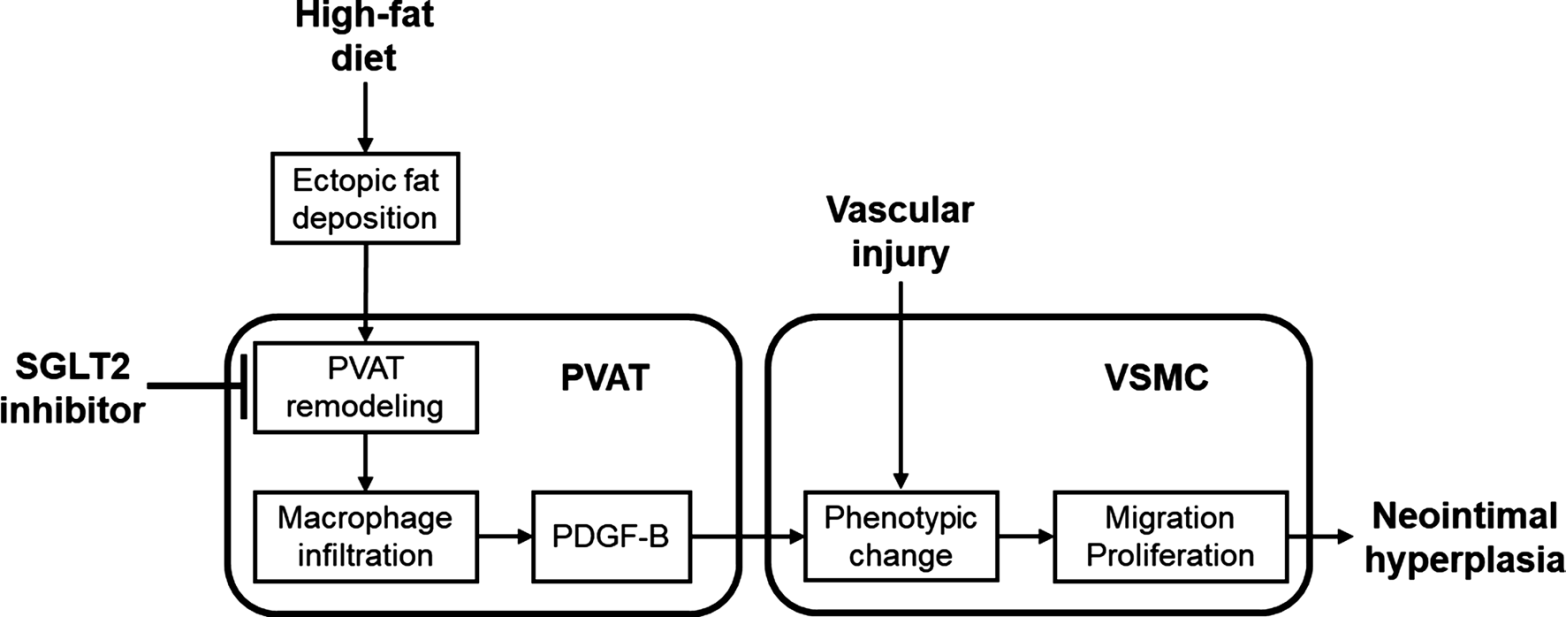
Proposed mechanisms for suppression of neointimal formation by luseogliflozin. High-fat diet-induced ectopic fat deposition leads to the PVAT remodeling and infiltration of PDGF-B-expressed macrophages, which may promote the neointimal hyperplasia after wire injury. *VSMC* vascular smooth muscle cells

In this study, we found that luseogliflozin significantly decreased the adipocyte size in PVAT surrounding the injured arteries of HFD-fed mice in association with increased gene expression of *adiponectin*. Moreover, PDGF-B-expressed macrophage infiltration into the PVAT was suppressed by the treatment with luseogliflozin. Gene expression of *Pdgf*-*b* in PVAT was correlated with I/M ratio, and effects of luseogliflozin on anthropometric and metabolic parameters in HFD-fed mice were modest in this study. Therefore, the present findings suggest that luseogliflozin could inhibit the PVAT remodeling and restore *adiponectin* expression, a marker of matured adipocytes [32, 33], thereby reducing the neointimal formation after wire injury via suppression of *Pdgf*-*b* expression. There is accumulating evidence that PDGF-B, a potent mitogen and migratory factor for vascular smooth muscle cells, was highly expressed at the site of balloon-injured arteries, thus being involved in the pathogenesis of neointimal hyperplasia after balloon injury [34–37]. Furthermore, PDGF-B is produced from macrophages infiltrated into VAT of obese mice [38]. These observations support our concept that suppression of PVAT remodeling and resultant inhibition of PDGF-B-expressed macrophage infiltration by luseogliflozin may play an important role in reducing the neointimal formation after wire injury in HFD-fed mice (Fig. 3). Given the anti-inflammatory effects of adiponectin on macrophage infiltration [39, 40], restoration of *adiponectin* expression may partly contribute to the suppression of PDGF-B-expressed macrophage infiltration into PVAT. Moreover, since adiponectin has reported to attenuate the action of PDGF-B in smooth muscle cells by inhibiting the association of PDGF-B with its receptor [41], PDGF-B-binding property of adiponectin may also be involved in suppression of neointimal formation.

In this study, luseogliflozin treatment caused some alterations in plasma lipid parameters; it significantly increased total cholesterol levels in HFD-fed mice and HDL-cholesterol in LFD-fed mice (Table 1), respectively, both of which were consistent with the previous findings in type 2 diabetic patients [42]. Furthermore, in contrast to the case of patients [42], luseogliflozin elevated triglycerides levels in LFD-fed mice.

### Limitations

There are several limitations in the present study. First, luseogliflozin treatment reduced the adipocyte size of PVAT surrounding femoral arteries. However, it did not affect that of epididymal adipose tissue, another type of VAT. VAT is exclusively composed of white adipose tissue, whereas PVAT has characteristic features of both white and brown adipose tissue [7, 8]. This may partly explain the different responses to luseogliflozin between epididymal adipose tissue and PVAT.

Second, luseogliflozin could inhibit vascular injury through various mechanisms; it may also act on smooth muscle cells and endothelial cells [43]. However, in this study, we found that PVAT removal significantly suppressed the neointimal hyperplasia in HFD-fed mice, whose I/M ratio was almost similar to that in LFD-fed mice without PVAT removal (I/M ratio of HFD-fed mice without PVAT, 0.99 ± 0.31; I/M ratio of LFD-fed mice without PVAT removal, 1.07 ± 0.54; *p* = 0.69). Furthermore, PVAT removal did not affect the neointimal hyperplasia in LFD-fed mice. These findings indicated that HFD-evoked deterioration of neointimal hyperplasia was totally dependent on PVAT. In addition, we also found here that luseogliflozin significantly suppressed the neointimal hyperplasia in HFD-fed mice, whose I/M ratio was almost similar to that in LFD-fed mice without PVAT removal (I/M ratio of luseogliflozin-treated HFD-fed mice, 1.06 ± 0.28; I/M ratio of LFD-fed mice without PVAT removal, 1.07 ± 0.54; *p* = 0.96). Moreover, luseogliflozin did not affect the neointimal hyperplasia in LFD-fed mice. So, the observations demonstrated that effect of luseogliflozin on neointimal hyperplasia was totally dependent on HFD. Therefore, given that luseogliflozin exerted no additive effects on neointimal hyperplasia in HFD-fed mice with PVAT removal, it is highly probable that luseogliflozin inhibited the neointimal hyperplasia in HFD-fed mice through the action on PVAT.

Third, although in this study, luseogliflozin treatment reduced PDGF-B-expressed macrophage infiltration into in remodeled PVAT, it remains unclear whether luceogliflozin could act on macrophages via SGLT2-dependent or -independent mechanisms [43].

Fourth, as far as we know, there are two reports to show that SGLT2 inhibitors suppressed the neointimal hyperplasia after arterial injury in rodent models [44, 45]. One study showed that ipragliflozin, an oral inhibitor of SGLT2 attenuated cuff-induced femoral artery remodeling in western diet-fed mice partly via increase in adipocyte size of abdominal PVAT with reduced secretion of pro-inflammatory factors [44]. On the other hand, ipragliflozin treatment did not show anti-inflammatory effects in thoracic PVAT, which were consistent with our present findings in femoral PVAT [44]. Effects of SGLT2 inhibitors on adipocytes may differ among the location of PVAT; howerver, we did not know the exact reason for the discrepant effects of SGLT2 inhibitors on adipocyte size and pro-inflammatory factors among abdominal, thoracic, and femoral PVAT. Furthermore, since the other study suggested the direct growth inhibitory effect of empagliflozin on smooth muscle cells [45], it would be interesting to clarify the effects of luceogliflozin on PDGF-B-induced smooth muscle cell proliferation and migration in vitro.

Fifth, in this study, we could not evaluate *adiponectin* and *pdgf*-*b* gene expression in femoral PVAT of LFD-fed mice because PVAT was barely detectable in LFD-fed mice and we were not able to obtain enough PVAT samples for RT-PCR analysis. Furthermore, the aim of this study was to clarify whether and how suppression of PVAT remodeling could protect against vascular injury in animal models. Since we found here that I/M ratio and adipocyte size were significantly smaller in LFD-fed mice compared with HFD-fed mice, PVAT derived from LFD-fed mice may not be suitable samples for analyzing the pathological association of PVAT remodeling with neointimal formation.

## Conclusions

Our present study suggests that luseogliflozin could attenuate the neointimal hyperplasia after wire injury in HFD-fed mice partly through the suppression of PVAT remodeling via inhibition of PDGF-B-expressed macrophage infiltration. Inhibition of PAVT remodeling by luseogliflozin may be a novel therapeutic target for vascular remodeling after balloon or stent angioplasty.

## Data Availability

The datasets used and/or analysed during the current study are available from the corresponding author on reasonable request.

## Abbreviations

CVD: cardiovascular disease
HbA1c: glycated hemoglobin
HDL: high-density lipoprotein
H&E: hematoxylin and eosin
HFD: high-fat diet
IL: interleukin
I/M: intima-to-media
LFD: low-fat diet
MCP: monocyte chemoattractant protein
PDGF: platelet-derived growth factor
PVAT: perivascular adipose tissue
RT-PCR: reverse-transcription polymerase chain reaction
SGLT: sodium-glucose cotransporter
TNF: tumor necrosis factor
VAT: visceral adipose tissue
VSMC: vascular smooth muscle cell
